# Human HMGA2 protein overexpressed in mice induces precursor T-cell lymphoblastic leukemia

**DOI:** 10.1038/bcj.2014.46

**Published:** 2014-07-11

**Authors:** A Efanov, N Zanesi, V Coppola, G Nuovo, B Bolon, D Wernicle-Jameson, A Lagana, A Hansjuerg, F Pichiorri, C M Croce

**Affiliations:** 1 Division of Hematology, College of Medicine, Comprehensive Cancer Center, The Ohio State University, Columbus, OH, USA; 2 Department of Molecular Virology, Immunology and Medical Genetics, Human Cancer Genetics Program, Comprehensive Cancer Center, OSU School of Medicine, The Ohio State University, Columbus, OH, USA; 3 Department of Pathology, Human Cancer Genetics Program, Comprehensive Cancer Center, OSU School of Medicine, The Ohio State University, Columbus, OH, USA; 4 Phylogeny Laboratory, Columbus, OH, USA; 5 Department of Veterinary Biosciences, Comparative Pathology and Mouse Phenotyping Shared Resource, Comprehensive Cancer Center, The Ohio State University, Columbus, OH, USA

## Abstract

T-cell acute lymphoblastic leukemia (T-ALL) is a neoplasia of thymocytes characterized by the rapid accumulation of the precursors of T lymphocytes. *HMGA2* (high-mobility group AT-hook 2) gene expression is extremely low in normal adult tissues, but it is overexpressed in many tumors. To identify the biological function of HMGA2, we generated transgenic mice carrying the human *HMGA2* gene under control of the V_H_ promoter/Eμ enhancer. Approximately 90% of Eμ-*HMGA2* transgenic mice became visibly sick between 4 and 8 months due to the onset and progression of a T-ALL-like disease. Characteristic features included severe alopecia (30% of mice); enlarged lymph nodes and spleen; and profound immunological abnormalities (altered cytokine levels, hypoimmunoglobulinemia) leading to reduced immune responsiveness. Immunophenotyping showed accumulation of CD5+CD4+, CD5+CD8+ or CD5+CD8+CD4+ T-cell populations in the spleens and bone marrow of sick animals. These findings show that HMGA2-driven leukemia in mice closely resembles spontaneous human T-ALL, indicating that *HMGA2* transgenic mice should serve as an important model for investigating basic mechanisms and potential new therapies of relevance to human T-ALL.

## Introduction

T-cell acute lymphoblastic leukemia (T-ALL) is an aggressive malignancy of T cells that arises mainly in children and adolescents.^1^ T-ALL accounts for 10% of pediatric and 25% of adult T-cell lymphoma cases, and it is more common among males than in females.^1^ Clinically, T-ALL patients show abnormal immune responses and levels of cytokines.^2^ It has also been reported that T-ALL patients develop severe hypoimmunoglobulinemia.^3^ Malignant transformation of developing thymocytes is a multistep process caused by genetic abnormalities that alter the normal mechanisms of cell growth control, proliferation and differentiation.^1^ The genetic hallmark of T-ALL is translocation and aberrant expression of one or more transcription factors, such as *TAL1, TAL2, LMO1, LMO2* and *HOX11L2*.^1^ NOTCH1 also has an important role in substantial proportion of T-ALL cases, with activating mutations of NOTCH1 present in about 50% of T-ALL cases examined.^1^ All activating mutations are located in the heterodimerization domain and/or proline/glutamic acid/serine/threonine PEST domain of NOTCH1.^4^ The majority of malignant T-ALL cells express CD5, CD7, CD4, CD8, CD2, CD45 and terminal deoxynucleotidyl transferase (TdT).^5^ About 78% of all T-ALL cases are CD4+CD8+, 17% are CD4−CD8− and 90% express TdT.^5^ T-ALL cases can be classified into well-defined groups based on gene expression profiling.^6, 7^ Examples of such groupings include TAL/LMO, HOHA, TLX, proliferative cluster (MKI67) and immature cluster.^6^ Each group shares a unique gene expression repertoire corresponding to distinct stages of T-cell development.^6, 7^

The high-mobility group (HMG) protein family is a class of non-histone chromosome-associated elements having common features such as being smaller than 30 kDa and having an abundance of acidic and basic amino acids.^8^ In mammals, HMG proteins consist of three families: HMGA, HMG1/2 and HMG14/17.^8^ HMGA2 is a member of the HMG AT-hook (HMGA) protein family, whose members possess DNA-binding activity to AT-rich regions through three basic domains (referred to as the ‘AT-hook'), located in the amino-terminal region.^9^ The carboxyl-terminal region of HMGA2 contains a highly acidic tail.^9^ HMG proteins, including HMGA2, cannot initiate transcription, but instead are involved in dynamic changes in DNA conformation and orchestrating the assembly of multicomponent nucleoprotein complexes that participate in gene replication and transcription.^8, 9^

Several studies report direct involvement of *HMGA2* locus chromosomal translocations in the pathogenesis of several types of leukemia such as B-cell chronic lymphocytic leukemia and acute myeloid leukemia,^10^ and one study has linked the rearrangement of the *HMGA2* locus with the development of T-ALL.^11^ It has also been demonstrated that human HMGA2 expressed in mice causes the onset of pituitary adenomas by enhancing E2F1 activity.^12^ Overexpression of the truncated form of human HMGA2, lacking the C-terminal tail, leads to the development of natural killer (NK) T-cell lymphoma in mice.^9^

To further clarify the biological role of human HMGA2, we generated a new mouse model carrying the wild-type (WT) human *HMGA2* under the control of the V_H_ promoter/Eμ enhancer, which drives specifically the expression of genes in B cells. Therefore, the expectation was that the transgenic (tg) mice would develop B-cell leukemia. However, in transgenic mice, the Eμ enhancer overexpresses linked reading frames in T cells as well,^13^ which led, in our *HMGA2* transgenic mice, to the onset and progression of T-cell leukemia with many traits similar to spontaneous human T-ALL. In this report, we describe the clinical, pathological, immunological and biochemical features of this new Eμ-*HMGA2* transgenic mouse model of T-ALL.

## Materials and methods

### Production of Eμ-*HMGA2* transgenic mice

A 373-bp fragment containing the human *HMGA2* open reading frame and 3′-HA was cloned into the EcoRV and SalI sites of the pBSVE6BK (pEμ) plasmid containing a mouse V_H_ promoter (V186.2), the immunoglobulin H (IgH)-Eμ enhancer and the poly(A) site of the human β-globin gene.^14^ The transgenic construct was cut out from vector sequences and injected into fertilized oocytes from FVB/N mice. Transgenic mice were produced in The Ohio State University transgenic mouse facility. Mice were screened for the presence of the transgene by PCR analysis of tail DNAs. Primers were:

Eμ-dir (37-MER) 5′-TGCTCATGAATATGCAAATCCTGTGTGTCTACAGTGG-3′

HMGA2 rev (30-MER) 5′-GGAGAGGGCTCACCGGTTGGTTCTTGCTGC-3′

Two male founders (F_0_ generation) were obtained (F9 and F28) and bred to wild-type FVB/N females. Transgenic progeny derived from these founders were studied and compared with non-transgenic siblings raised in identical conditions. The animal studies (The Ohio State University protocol 2010A00000146) were approved by The Ohio State University Institutional Animal Care and Use Committee and were conducted under National Institutes of Health guidelines.

### Phenotypic analysis of Eμ-*HMGA2* transgenic mice

Young adult mice were necropsied to define macroscopic changes, obtain whole blood for hematologic analysis and collect selected tissues for histopathologic evaluation. Organs were fixed in neutral buffered 10% formalin and processed into paraffin using conventional methods. Lesions were evaluated in sections stained with hematoxylin and eosin. For selected neoplastic foci, serial sections were stained and labeled by indirect immunoperoxidase histochemistry to demonstrate the distribution of B cells and T cells within tissues.

### Western blot analysis and mice immunization

Proteins from spleens were extracted with Nonidet P-40 lysis buffer as previously described.^15^ HMGA2 expression was detected in western blots using an HA antibody. Actin-b staining was done to verify equivalent protein loading. Mice, 4–8 months old, were immunized intravenously with 120 μg of keyhole limpet hemocyanin (KHL; Life Diagnostics, West Chester, PA, USA) to elicit a KHL-specific antibody immune response. Pre-immune blood samples were obtained 2 days before immunization, whereas post-immune blood samples were drawn and analyzed 5 days after immunization. Serum levels of anti-KHL antibodies were measured using an enzyme-linked immunoassay kit (Life Diagnostics) according to the manufacturer's instructions. Briefly, 100 μl standards and mouse serum were loaded to each well of a KHL-coated 96-well plate, incubated for 45 min at room temperature (RT) and washed five times with wash solutions. After washing, 100 μl of peroxidase-labeled anti-KHL antibody conjugate was added to each well, incubated for 45 min at RT and washed five times. For visualization of the antigen–antibody reaction, the substrate 3,3′,5,5′-tetramethylbenzidine was added 100 μl per well, and plates were left at RT for 20 min. To avoid an increase in the background, the reaction was stopped by adding 100 μl per well of 1 M HCl.

### Isolation and immunophenotyping of lymphocytes from lymphoid organs

Lymphocytes from spleens and lymph nodes were isolated as previously described.^15^ T cells and B cells were isolated using a B-Cell Isolation Kit (Miltenyi Biotec, Auburn, CA, USA) according to the manufacturer's instructions. For immunostaining, a single-cell suspension was prepared in phosphate-buffered saline supplemented with 1% fetal bovine serum (‘staining solution'). Cells were washed in this solution and then incubated with antibodies for 15 min at RT. Flow cytometry was carried out on a LSR II flow cytometer using BD FACSDiva software, and on a FACSCalibur using CellQuestPro software (all instruments and software were provided by BD Biosciences, San Jose, CA, USA). Conjugated monoclonal antibodies directed against CD5(53-7.3), CD19(1D3), CD23(B3B4), B220(RA3-6B2), CD4(GK1.5), CD8(53-6.7) and CD3(SP34-2) were purchased from BD Biosciences and used according to the manufacturer's instructions.

### Measurements of serum immunoglobulin and cytokines levels

Immunoglobulin levels were measured by using the Milliplex map Mouse Immunoglobulin Isotyping kit (EMD Millipore Corporation, Billerica, MA, USA) according to the manufacturer's instructions. Briefly, 50 μl of standards and mice sera were loaded to each well of a 96-well plate, after which 25 μl of solution containing beads capable of binding IgA, IgG1, IgG2a, IgG2B or IgG3 were added to each well. The plate was incubated for 15 min with agitation on a plate shaker at RT. After incubation, wells were washed two times with wash buffer, then 25 μl of anti-mouse k-light-chain antibody conjugated with phycoerythrin was added. The plate was incubated for 15 min with agitation on a plate shaker at RT. Next, the wells were washed two times with wash buffer and read in a MAGPIX instrument (Luminex Corporation, Madison, WI, USA). The quantity of immunoglobulins was calculated from the standard curve constructed from standards, and corrected for the serum dilutions. Cytokine levels in serum were measured using a Mouse Inflammation Kit (BD Biosciences) according to the manufacturer's instructions. We determined the levels of interleukin (IL)-2, IL-10, interferon gamma, IL-12p70, monocyte chemoattractant protein-1 and tumor necrosis factor alpha.

### Proliferation of CD5+CD4+ lymphocytes

One day before analysis, 1 mg of 5-bromo-2′-deoxyuridine (BrdU) was injected into the peritoneum of *HMGA2* transgenic mice and wild-type control mice. The day of necropsy, lymphocytes from spleens were collected, and proliferation in the population of CD5+CD4+ lymphocytes was measured using the BrdU Flow Kit (BD Biosciences) according to the manufacturer's instructions.

### Sequence analysis

The heterodimerization and PEST domains of Notch1 were amplified using primers described elsewhere.^4^ TAL2 was amplified with primers:

DIR: 5′-ATGACCAGAAAGATCTTCACAAATACC-3′

REV: 5′-TTAGGGAGCCCCGTGGCTTGGGCCAGG-3′

PCR products were purified using the Promega Quick Purification kit (Promega Corporation, Madison, WI, USA) and sequenced. The sequence was compared with wild-type *NOTCH1* (GeneBank accession number: NM_008714) and *TAL2* (GeneBank accession number: M81077.1).

## Results

**Figure 1:**
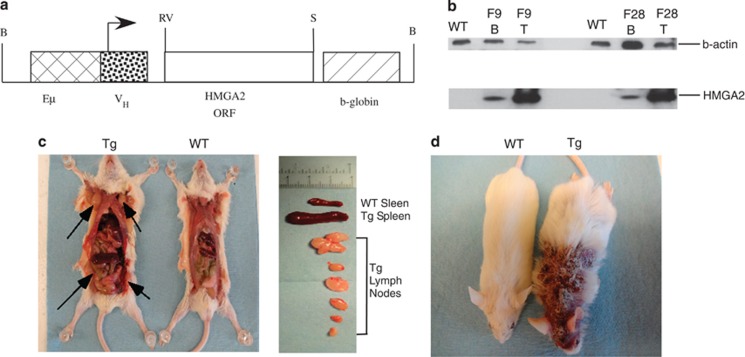
(**a**) Eμ-*HMGA2* construct. (**b**) HMGA2 expression in B and T cells of two transgenic lines. Western blot analysis was carried out using anti-HA antibodies. WT spleen lysate from a wild-type FVB/N mouse. F9 and F28 spleen lysates from F9 and F28 transgenic lines correspondingly. (**c**) Gross pathology of a representative 5-month-old Eμ-*HMGA2* transgenic mouse, exhibiting greatly enlarged lymph nodes (arrows) and spleen relative to a wild-type of the same age. (**d**) A representative Eμ-*HMGA2* transgenic mouse with severe skin lesion (right) and wild-type counterpart (left).

We generated transgenic mice bearing the human *HMGA2* gene with 3′-HA under the control of the V_H_ promoter/Eμ enhancer with the poly(A) site of the human globin gene (Figure 1a). The Eμ enhancer specifically drives expression in B cells, but also is active in T cells.^13^ We selected two founders and entitled them F9 and F28. These founders were bred with wild-type FVB/N mice to establish two transgenic lines. Expression of HMGA2 was determined by western blot in B- and T-splenocyte protein lysates using anti-HA antibody. Figure 1b shows high HMGA2 expression in T cells of both lines and moderate expression in B cells.

Approximately 90% of *HMGA2* transgenic mice became visibly sick at 4–8 months of age, whereas age-matched wild-type controls remained healthy. Nearly all *HMGA2* transgenic animals developed enlarged spleens and multiple enlarged lymph nodes (Figure 1c). About 30% of *HMGA2* transgenic mice also presented with severe alopecia (Figure 1d) associated with a maculopapular skin lesion (Figure 3j).

**Figure 2:**
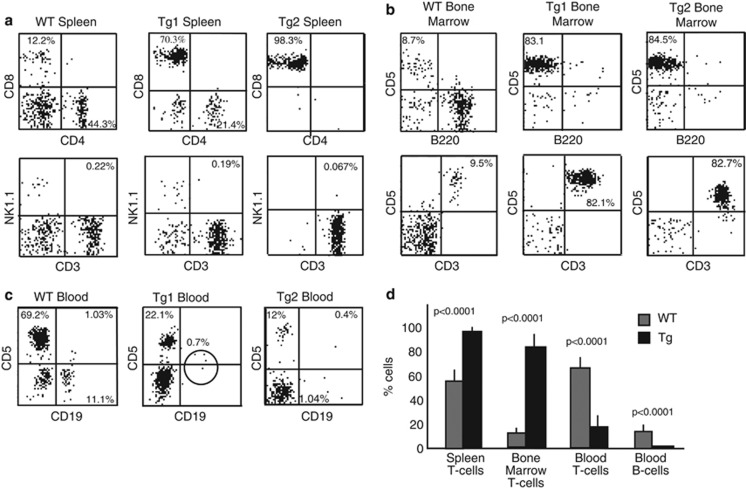
(**a**) Flow cytometric analysis of Eμ-*HMGA2* transgenic mice and wild-type control lymphocytes isolated from (**a**) spleen, (**b**) bone marrow and (**c**) peripheral blood. Representative images for five mice per genotype. (**d**) Population frequencies of transgenic and age-matched wild lymphocytes isolated from spleen, bone marrow and peripheral blood.

We used flow cytometry to evaluate the immunophenotype of spleen, bone marrow and blood cells from *HMGA2* transgenic mice. At 4–8 months of age, flow cytometric analysis showed that nearly all splenic cells from transgenic animals were T cells (Figure 2a, upper right). We analyzed 21 transgenic animals, which became visibly sick, and discovered that 95±3.3% of splenic cells were T cells versus 58±8.6% (*n*=5, *P*<0.0001) of T cells in wild-type littermates. These findings indicate that our mice develop T-cell leukemia. Figure 2b shows two representative examples from the transgenic animals (upper right) and a wild-type counterpart (upper left). To check whether *HMGA2* transgenic mice developed NK/T-NK lymphomas, we performed CD3/NK1.1 double staining of transgenic and age-matched wild-type splenic cells. Flow cytometric analysis revealed no significant difference between the CD3+NK1.1+ cell populations in the transgenic and wild-type animals (0.18±0.05%, *n*=21 versus 0.2±0.06%, *n*=5, *P*=0.44) as shown in Figure 2a (bottom).

As we found that the T-cell population in the spleens of *HMGA2* transgenic mice was markedly expanded in comparison with wild-type mice, we decided to also evaluate the T-cell population in the blood and bone marrow of transgenic animals. Flow cytometric examination of transgenic and wild-type bone marrow revealed that 82.7±10.3% (*n*=5) of bone marrow lymphocytes were T cells versus 9.1±2.5% (*n*=5, *P*<0.0001) of those cells in age-matched wild type littermates (Figures 2b and d). Surprisingly, blood of transgenic mice contained fewer T cells (17.1±7.5%, *n*=5, *P*<0.0001) relative to wild-type mice (65.3±7.1%, *n*=5), and transgenic animals also had essentially no circulating B cells (0.6±0.2% B cells in Tg versus 12.4±3.5% WT, *n*=5, *P*<0.0001; Figures 2c and d).

**Figure 3:**
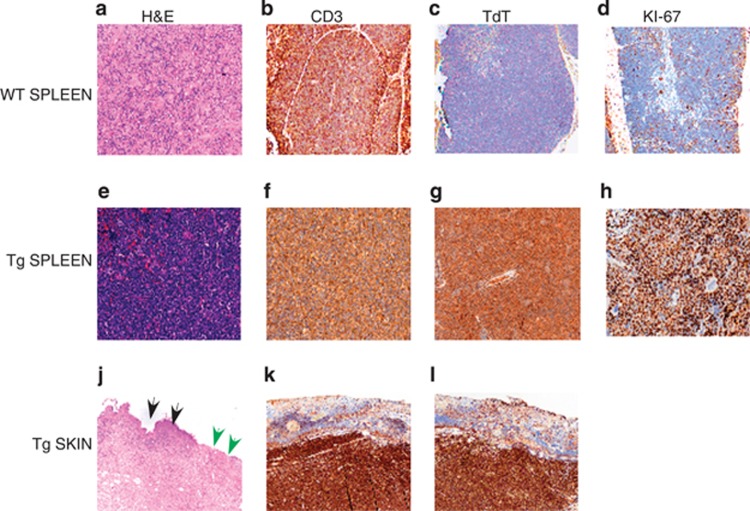
Histopathological findings of adult Eμ-*HMGA2* mice and wild-type control mice. The upper row demonstrates the spleen from a representative wild-type animal, using serial sections stained with hematoxylin and eosin (H&E) or labeled by immunohistochemistry (IHC) to reveal lymphocytes (the CD3 marker), TdT-positive cells and proliferating cells (Ki-67), whereas the middle row shows corresponding regions from the spleen of a representative HMGA2 transgenic mouse. The transgenic animal clearly exhibits a diffuse infiltration by myriad, proliferating neoplastic lymphocytes. The lower row shows the ulcerated skin from a transgenic mouse, indicating both ulcer (green arrows) and intact skin (black arrows). (**a**, **e**, **j**) Routine hematoxylin and eosin staining of WT splenic tissues, Tg splenic tissues and Tg skin lesions, respectively. (**b**, **f**, **k**) CD3 immunohistochemistry of WT splenic tissues, Tg splenic tissues and Tg skin lesions, respectively. (**c**, **g**, **l**) TdT staining of WT splenic tissues, Tg splenic tissues and Tg skin lesions, respectively. (**d**, **h**) KI-67 staining of WT splenic tissues and Tg splenic tissues, respectively.

To further confirm that Eμ-*HMGA2* transgenic mice develop T-ALL-like disease, we carried out histological and immunohistological analysis of lymphoid tissues. Figures 3a and e show the routine hematoxylin and eosin findings in the WT versus Tg splenic tissues. These pictures indicate the relatively low cell density typical of the wild-type spleen, whereas the transgenic spleen had a much greater cell density evident as a dark blue color typical of a neoplastic mononuclear infiltrate. The CD3 immunohistochemistry for T cells showed that about 50% of the cells in the WT spleen were positive, whereas in the Tg spleen over 90% of the cells in the infiltrate were labeled by this marker (Figures 3b and f). Figures 3c and g show the TdT staining of the WT and Tg splenic sections showed the absence of TdT in the WT spleen (Figure 3c), whereas most of the mononuclear cells in the Tg spleen was positive for TdT, which is diagnostic of the T-ALL in mice.^16^ The Ki-67 stain for the WT spleen shows the expected occasional positive cells for this proliferation marker (Figure 3d), whereas in the Tg spleen nearly 100% of cells expressed Ki-67 (Figure 3h); this very high proliferation index is characteristic of an aggressive, rapidly growing leukemia. The hematoxylin and eosin findings of the transgenic skin showed that the dermis was markedly expanded by a broadband infiltrate of mononuclear cells; the infiltrate also involves the subcutaneous adipose tissue (Figure 3j). The skin near the margins of ulcers often exhibited small elevations due to the dense population of underlying tumor cells (that is, maculopapular skin lesion). Figure 3j shows that the overlying epithelium is lost on the right side of the picture (green arrowheads) due to the ulceration of the skin by the infiltrate. The intact skin is marked by black arrowheads. All of the cells in the infiltrate under the ulcer showed intense staining with CD3 and TdT (Figures 3k and f), which is indicative of T-ALL. Taken together, these findings clearly indicate that Eμ-*HMGA2* transgenic mice develop acute T-cell lymphoblastic leukemia.

**Figure 4:**
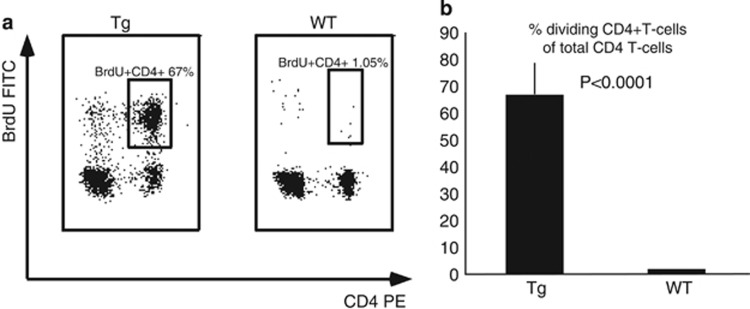
(**a**) Proliferation of malignant T cells from Eμ-*HMGA2* transgenic mice and normal T cells from wild-type control animals. The numbers indicate the frequency of CD4+ population. Representative images for five mice per genotype. (**b**) Proliferation rate of transgenic and wild type splenocytes.

The T-ALL neoplastic cells are actively proliferating.^17^ The majority (67±9.4%, *n*=5) of splenic T cells from HMGA2 transgenic animals but essentially no cells (1.05±0.19%, *n*=5, *P*<0.0001) from age-matched wild-type mice exhibited substantial BrdU incorporation (Figures 4a and b), showing that T cells in the transgenic mice were proliferating robustly.

**Figure 5:**
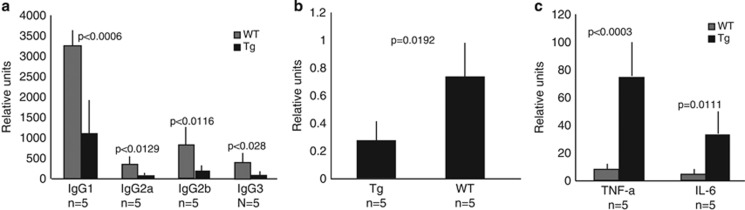
(**a**) The levels of immunoglobulins in 5–8-month-old Eμ-*HMGA2* transgenic mice are much lower to those of age-matched wild-type control mice. (**b**) Production of anti-KHL antibodies as indicated by circulating serum levels in transgenic mice was lower than that of wild-type counterparts. (**c**) The levels of tumor necrosis factor alpha and IL-6 in serum of Eμ-*HMGA2* transgenic mice were greatly elevated relative to those of their wild-type littermates.

Human T-ALL is characterized by immune incompetence, abnormal cytokine production and severe hypogammaglobulinemia.^2, 3^ Serum levels of immunoglobulins in Eμ-*HMGA2* transgenic mice were severely decreased relative to those in wild-type littermates (Figure 5a), including IgG1, IgG2a, IgG2b and IgG3. The levels of IgG1 were decreased by about threefold, and the levels of IgG2a, IgG2b and IgG3 by 3.5-fold. In contrast, there was no difference in IgA levels. The ability of HMGA2 transgenic mice to mount an immune response to an antigen KHL was reduced by about threefold in comparison with wild-type counterparts (Figure 5b). Serum levels of IL-6 were sixfold higher and those of tumor necrosis factor alpha levels were 10-fold higher in *HMGA2* transgenic animals compared with wild-type counterparts (Figure 5c).

Because the mutations of gene *NOTCH1* have been often observed in human T-ALL, we decided to check the mutational status of NOTCH1 in our transgenic model. We analyzed 10 tumor samples of *HMGA2* transgenic animals and no mutation were found (data not shown).

We have also checked the mutational status of TAL2 locus because its aberrant expression is linked with T-ALL development and no mutations were detected (data not shown).

## Discussion

Our two lines of Eμ-*HMGA2* transgenic mice carrying the human *HMGA2* gene under the control of the V_H_ promoter/Eμ enhancer both developed lymphoproliferative disease bearing a strong resemblance to human T-ALL. The result is somewhat unexpected as the Eμ enhancer is considered to be a B-cell-specific enhancer, but it also is able to drive the expression of linked gene(s) in T cells.^13^ About 90% of Eμ-*HMGA2* transgenic mice presented with enlarged spleens and lymph nodes between 5 and 8 months of age. Histological and fluorescence-activated cell sorting analyses of splenocytes from transgenic animals revealed that malignant cells were immature T-cells expressing TdT and lacking NK1.1, which is a common immunophenotype of T-ALL in human patients. At first glance, this result is a little surprising taking into consideration that transgenic mice bearing human *HMGA2* under the transcriptional control of cytomegalovirus (CMV) promoter have been reported to mainly develop pituitary adenomas.^18^ One possible explanation for this discrepancy is variations in the level of *HMGA2* expression. The expression of *HMGA2* in the CMV-*HMGA2* mouse model was checked by reverse transcription PCR,^18^ but this method demonstrates the relative amount of *HMGA2* mRNA, not the actual level of HMGA2 protein. In addition, the levels of *HMGA2* mRNA in the CMV-*HMGA2* model were not measured separately in B cells and T cells. In spite of the relatively high level of *HMGA2* mRNA presented in spleens of CMV-*HMGA2* transgenic mice, the actual levels of HMGA2 protein in splenic B cells and T cells or in T cells alone could be lower than the threshold concentration required to exert an oncogenic effect in lymphocytes. Another factor to take into consideration is the different genetic backgrounds of Eμ-*HMGA2* (FVB/N) and CMV-*HMGA2* (B6C3) models, which could have permitted divergent phenotyping manifestations. It has been reported that overexpression of human *HMGA2* deprived of the C-terminal acidic tail caused NK cell lymphomas in mice with a penetration of 35% by 12 months of age (CMV-*HMGA2* truncated).^9^ This mouse model was generated on C57B6/J background using embryonic stem cell-mediated strategy, and *HMGA2* expression was controlled by the CMV promoter.^19^ The embryonic stem cell approach allows a very high, often supraphysiological expression of a transgene, thus causing changes that are not physiologically relevant effects.^18^ Interestingly, when CMV-*HMGA2*-truncated transgenic mice were generated by microinjection of the same construct into mouse zygotes, the levels of *HMGA2* expression were much lower than those in mice generated with the embryonic stem cell-mediated approach, which also resulted in a much lower penetrance and more advanced age of onset for the NK/T-NK lymphomas.^9^

Abnormal cytokine production, immune incompetence, an extensive proliferation rate and hypogammaglobulinemia are important features of T-ALL in human patients.^2, 3, 17^ We showed that Eμ-*HMGA2* transgenic mice also have hypogammaglobulinemia, a threefold decrease in the immune response against the foreign antigen KHL, and elevated levels of the cytokines' tumor necrosis factor alpha and IL-6. We also confirmed the high proliferation rate of malignant cells by using a BrdU incorporation assay. Therefore, the Eμ-*HMGA2* transgenic mouse model shares the same functional attributes and molecular signature as human T-ALL.

Skin lesions are not characteristic of human T-ALL, but about 30% of Eμ-*HMGA2* transgenic mice developed severe alopecia with typical maculopapular skin lesions (Figure 3j). Histological examination of these skin lesions revealed that subcutaneous regions were densely populated by malignant T cells (Figures 3k and l). Thus, *HMGA2* transgenic mice could serve as a model for human maculopapular skin lesion.

Because HMGA2 overexpressed in mice leads to T-ALL development, we decided to check the level of *HMGA2* expression in three different subsets of human T-ALL-TAL/LMO, HOXA and TLX using publically available Gene Expression Omnibus database (http://www.ncbi.nlm.nih.gov/geo/). Normal human bone marrow was used as a control.^6^ The lowest level of *HMGA2* expression was detected in normal human bone marrow, but the difference between normal human bone marrow and the T-ALL subsets was not substantial, indicating that either HMGA2 did not have an essential role in the development of the cohort of T-ALL cases analyzed^6^ or that the level of *HMGA2* transcripts did not reflect the actual level of HMGA2 protein. Because NOTCH1 signaling has an important role in T-ALL development, we have evaluated the mutational status of heterodimerization and PEST domains of *NOTCH1*. We did not find any mutations in these domains. We also sequenced TAL2 oncogene and found no mutations in this gene. We used the Gene Expression Omnibus databases to check the levels of *HMGA2* mRNA *in vitro* model systems in which NOTCH1 is downregulated or upregulated in T-ALL cell lines to understand whether or not HMGA2 is a NOTCH1 target. Our Gene Expression Omnibus database data analysis showed that downregulation of NOTCH1 in some T-ALL cell lines caused the induction of HMGA2 expression, whereas upregulation of NOTCH1 led to the inhibition of HMGA2 at the transcriptional level. These data suggest that NOTCH1 signaling has no essential role in our transgenic model. We performed a search in ChIPbase (http://deepbase.sysu.edu.cn/chipbase) and found that several important T-ALL transcriptional factors such as c-myc and CDX2 bind to the promoter of *HMGA2*, suggesting that they could upregulate HMGA2 expression in some human T-ALL cases.^20, 21^

We found only one published report that linked the rearrangement of the *HMGA2* gene to T-ALL development.^11^ It would be very interesting to evaluate the levels of *HMGA2* protein expression in different T-ALL subsets and samples, to check *HMGA2* locus for mutations and rearrangements. Future investigations should be addressed to these questions. In summary, our results demonstrated that expression of the human *HMGA2* gene in mouse T cells led to T-ALL development. This is the first report linking the expression of full-length human *HMGA2* in mice with the initiation and progression of a particular cancer. Our novel T-ALL mouse model represents an important new tool for better understanding the molecular targets of HMGA2 in T cells, uncovering signaling pathways in which HMGA2 participates, and for testing potential anti-T-ALL drugs.
